# Mastadenovirus Molecular Diversity in Waste and Environmental Waters from the Lisbon Metropolitan Area

**DOI:** 10.3390/microorganisms10122443

**Published:** 2022-12-10

**Authors:** Joana Cavadas, Ricardo Parreira, Inês Leonardo, Maria Teresa Barreto Crespo, Mónica Nunes

**Affiliations:** 1 Instituto de Biologia Experimental e Tecnológica (iBET), Apartado 12, 2781-901 Oeiras, Portugal; 2 Unidade de Microbiologia Médica, Instituto de Higiene e Medicina Tropical (IHMT), Universidade Nova de Lisboa (NOVA), Rua da Junqueira No. 100, 1349-008 Lisboa, Portugal; 3 Global Health and Tropical Medicine (GHTM) Research Centre, 1349-008 Lisboa, Portugal; 4 Instituto de Tecnologia Química e Biológica António Xavier (ITQB), Universidade Nova de Lisboa (NOVA), Av. da República, 2780-157 Oeiras, Portugal

**Keywords:** human adenovirus, molecular diversity, wastewater, environmental waters, next-generation sequencing, nested PCR, phylogenetic analysis

## Abstract

In face of the absence of epidemiological data regarding the circulation of human adenoviruses (HAdV) in Portugal, this study aimed at the evaluation of their molecular diversity in waste and environmental waters in the Lisbon Metropolitan Area (LMA). Using samples collected between 2018 and 2021, the HAdV hexon protein-coding sequence was partially amplified using three nested touch-down PCR protocols. The amplification products obtained were analyzed in parallel by two approaches: molecular cloning followed by Sanger sequencing and Next-Generation Sequencing (NGS) using Illumina^®^ sequencing. The analysis of NGS-generated data allowed the identification of a higher diversity of HAdV-A (19%), -B (1%), -C (3%), -D (24%), and -F (25%) viral types, along with murine adenovirus (MAdV-2; 30%) in the wastewater treatment plant samples. On the other hand, HAdV-A (19%), -D (32%), and -F (36%) were identified in environmental samples, and possibly MAdV-2 (14%). These results demonstrate the presence of fecal contamination in environmental waters and the assessment of the diversity of this virus provides important information regarding the distribution of HAdV in LMA, including the detection of HAdV-F41, the most frequently reported in water worldwide.

## 1. Introduction

Around the world, several disease outbreaks associated with the presence of waterborne pathogens have been reported [1], as many pathogenic agents responsible for numerous infectious diseases can be transmitted by contact with, or consumption of, water or water-contaminated food items.

The main cause of fecal pollution in the aquatic environment is the discharge of both raw and partially treated sewage [2,3]. At wastewater treatments plants (WWTPs), such discharges might be the consequence of high inflows from rain or other infiltration, insufficient holding/storage capacity, equipment malfunction, or careless maintenance [4]. High-income countries usually provide treatment to approximately 70% of the wastewater generated, while upper- and lower middle-income countries treat around 40% and 30% of the wastewater generated, respectively. On the other hand, low-income countries treat less than 10% of total volume of wastewater generated, the rest being disposed of directly untreated into water bodies, such as rivers, lakes, and the ocean [5,6]. The effect that wastewater discharges have on watercourses and human and animal health depends on the volume of the discharge. In order to reduce the potential damage that the discharge may have, dilution is the key intervening component [7]. Considering that wastewater contains potentially harmful chemicals and pathogenic microorganisms, its unsupervised, direct discharge in the environment can also negatively affect aquatic habitats, changing species composition, and contributing to a decrease in biodiversity [6]. In addition, other important biological parameters that can change due to sewage disposal include the occurrence and/or introduction of enteric viruses [8].

Some of the waterborne pathogenic agents can remain in the water at high levels despite the treatments used in WWTPs [2,9,10], therefore representing a high risk to human populations. Among these agents, viruses are considered the most concerning group of pathogens found in wastewater, as they often remain infectious for long periods, and occur in higher concentrations, sometimes throughout the year [11]. For these reasons, it is not safe to depend only on bacteriological standards to evaluate water quality [12,13,14], as they underestimate the public health risk that virus-contaminated water may impose on human health [3]. As a result, the use of a viral indicator of fecal water contamination has been previously proposed [9,13]. Human adenoviruses (HAdV) stand out as a good candidate for this purpose due to their high resistance to water treatment and disinfection processes (especially when compared to the bacterial indicators), their high prevalence in all geographic areas, and host-specificity [9,13,14,15,16].

The International Committee for the Taxonomy of Viruses (ICTV) recognizes 87 Adenoviruses (AdV) species and six genera within the *Adenoviridae* family [17]. Within the *Mastadenovirus* genus AdVs infect a wide range of mammalian species including humans, bovines, murines, and non-human primates [18]. The AdV virions correspond to medium-sized (90–100 nm) non-enveloped infectious particles and are composed of an external icosahedral capsid and an internal core that encloses a linear double-stranded desoxyribonucleic acid (DNA) genome [19]. The capsid is built by self-assembly of, among others, the so-called hexon proteins, which form the icosahedron sides of the virion, and accounts for 60% of the viral particle’s total mass [20].

In particular, HAdVs are categorized into seven species (A through G) within the *Mastadenovirus* genus based on their physical, chemical, genetic, and biological properties [21,22]. Historically, different HAdV serotypes have been defined by classical methods such as viral neutralization assays, and hemagglutination properties. However, currently, they are mostly classified into genotypes by sequence analysis [19]. So, beyond the initial 51 recognized serotypes, more than 100 genotypes have already been described (http://hadvwg.gmu.edu/ accessed on 20 September 2022). Since the HAdV nomenclature has not reached a global consensus within the scientific community, the format “human adenovirus dash species type” (e.g., HAdV-C5) will be used in this work.

The different HAdV types do not exhibit the same tissue tropism and this is correlated with the heterogeneity of the clinical manifestations they cause [19,23]. These manifestations range from mild and self-limiting infections [19,23], which involve the upper or lower respiratory tract, gastrointestinal tract, or conjunctiva [24]. Although rare, severe manifestations such as hemorrhagic cystitis and colitis, hepatitis, pancreatitis, nephritis, or meningoencephalitis can also occur [19]. Vulnerable populations (children, elderly, and immunocompromised patients) have a higher risk of developing the severe disease [19,24] and, in some cases, infections may be fatal [21,23,25]. Of all seven species, HAdV-A, -F, and -G viruses have selective tropism for the gastrointestinal tract [23,26]. The HAdV-B species can cause respiratory and urinary tract infections [19], and the HAdV-C species are normally associated with acute respiratory tract infections in children. The HAdV-D species is mainly responsible for moderate clinical diseases or asymptomatic infections, frequently involving the gastrointestinal tract, except for a few types (HAdV-D8, -D37, -D53, -D54, -D64) that are responsible for epidemic keratoconjunctivitis [19,27,28]. The HAdV-E species is usually involved in outbreaks of acute respiratory disease in military units [19], but can also be responsible for conjunctivitis.

While the most common transmission route for HAdV involves the respiratory tract [22], all types (enteric and non-enteric) can be excreted in feces from both symptomatic and asymptomatic infected individuals [3,24,29]. As a result, these viruses can also be transmitted by the fecal–oral route [19,25] via ingestion of contaminated food/water and by contact with contaminated water.

HAdV have been widely detected in wastewater (influent and effluent), in marine and freshwater, as well as in treated and disinfected drinking water, tap water, and swimming pools [1,12,13,14,29,30,31,32,33,34,35,36,37,38,39,40]. Under these circumstances, it is important to monitor the disinfection efficacy of WWTP processes to evaluate the risks that the discharge of HAdV-contaminated effluent into watercourses has on public health. These watercourses, which receive treated wastewater, often provide drinking water and can be used for recreational activities, irrigation of parks, and sports fields, as well as in agricultural practices [16]. Consequently, if HAdV are introduced in an aquatic environment where they may remain infectious, human communities may end up being exposed to them. Additionally, according to Farkas et al. [41], HAdV may indicate the persistence of enteric viruses, so the adequate control of HAdV in water may suggest that other enteric viruses are also controlled [11].

In Portugal, epidemiological data on HAdV is scarce, with inconsistent data reporting their geographical distribution or prevalence, with only a few studies reporting their presence [42,43,44,45]. Therefore, the main purpose of this work regarded the evaluation of the molecular diversity of HAdV in raw sewage (i.e., influent) and environmental water samples collected within the Lisbon Metropolitan Area (LMA).

## 2. Materials and Methods

### 2.1. Sample Collection

**Figure 1:**
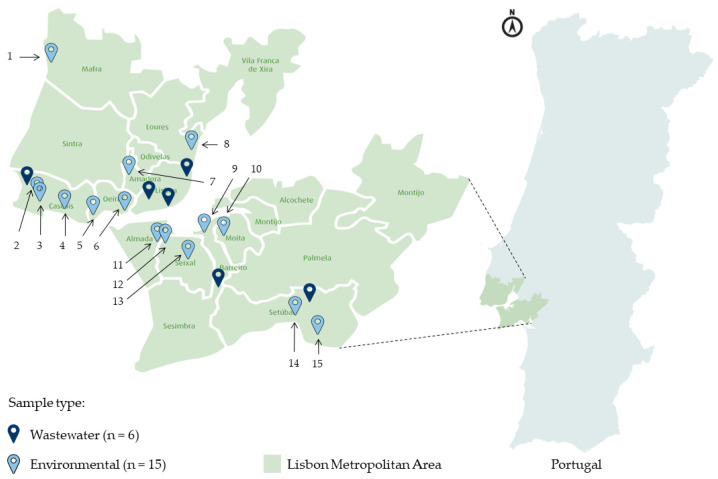
Geographic localization of the wastewater and environmental sampling sites in LMA. The total number of collection sites is indicated between brackets (bottom, right). The geographical coordinates of each environmental site collection are: 1 (38°56′24″ N 9°24′47″ W); 2 (38°42′55″ N 9°25′39″ W); 3 (38°43′6″ N 9°25′51″ W); 4 (38°41′42″ N 9°22′32″ W); 5 (38°41′9″ N 9°18′51″ W); 6 (38°41′41″ N 9°13′48″ W); 7 (38°45′24″ N 9°14′55″ W); 8 (38°47′47″ N 9°5′57″ W); 9 (38°39′55″ N 9°4′39″W); 10 (38°39′12.7″ N 9° 1′57″ W); 11 (38°38′38.6″ N 9°10′23.9″ W); 12 (38°38′26″ N 9°9′25″ W); 13 (38°36′45.8″ N 9°6′25.7″ W); 14 (38°31′14″ N 8°53′14″ W); 15 (38°30′22″ N 8°50′52″ W).

A total of 15 environmental and nine wastewater samples were collected at 21 different locations in LMA (Figure 1). For the wastewater samples, the solid particle-free influent (1 L) from six different WWTPs (A through F) were collected in October 2018 and April 2019, and in July, October, and November 2020, with some of the sites being sampled more than once. Owing to a confidentiality agreement, the localization and identification of the WWTPs studied cannot be revealed. The environmental water samples (10 L) were collected in October/November 2020, and October 2021. Six of the samples were collected from rivers, four from creeks, and five from ditches. All the environmental collection sites were selected based on their proximity to possible fecal pollution sources, including the vicinity of WWTPs and places where sewage discharges were known to occur, identified by residents’ complaints. All samples were brought to the laboratory at room temperature, immediately stored at 4 °C, and processed the day after collection.

### 2.2. Viral Particles Concentration by Skimmed Milk Flocculation, and DNA Extraction

The viral-like particles (VLP) were concentrated by skimmed milk flocculation, as previously described [46]. In brief, a pre-flocculated 1% (*w*/*v*) powdered milk solution was prepared using skimmed milk powder (Conda Pronadisa, Spain) dissolved in synthetic seawater (Paragon Scientific Ltd., Prenton, UK). Then, this solution was directly added to each sample to obtain a final concentration of skimmed milk of 0.01% (*w*/*v*). The pH of the solution was adjusted to 3.5 by adding HCl 1 M, and all the samples were subsequently stirred for 8 h at room temperature and then incubated for an additional 8 h at room temperature without agitation. The supernatant was then carefully removed with a vacuum pump, and the sediment was concentrated by centrifugation at 5500× *g* for 45 min at 12 °C. Finally, the concentrate was resuspended in 8 mL of phosphate buffer (1:2, *v/v* of 0.2 M Na_2_HPO_4_, 0.2 M NaH_2_PO_4_, pH 7.5), and stored at −80 °C until total DNA was extracted from these VLP concentrates using the QIAamp^®^ DNA Mini Kit (Qiagen, Hilden, Germany) [1] following the manufacturer’s instructions.

### 2.3. Primers Design

The primers used in this work were designed based on multiple sequence alignments starting from datasets that were constructed by compiling HAdV hexon nucleotide coding sequences corresponding to the viral types described [28]. The length of the sequence (>600 base pairs (bp)) and their geographic region of origin (preferably those previously described in Southern Europe and Portuguese-speaking African countries) were used as minimal inclusion criteria. Additionally, rarely represented viral types for which <6 sequences were available in the public nucleotide sequence databases (some HAdV-D and -G types) were not included.

The hexon-coding viral sequences were aligned using the iterative G-INS-I method implemented in MAFFT (version 7) [47] and edited with Gblocks [48]. Then, a phylogenetic tree was constructed by the neighbor-joining method using Mega software (version 6), and genetic distances were corrected with the Kimura-2P formula.

The great diversity of the hexon-coding sequences under analysis compromised the intended primer design when all sequences were tentatively aligned together. Therefore, from the initial dataset, three secondary datasets were prepared (HAdV (A + F), HAdV (B + E), and HAdV (D)), considering the amplification and detection of only the viral types found in Europe.

The primers were designed using Primer Design-M [49] considering default settings for multiple parameters such as their length, detection limit, maximum temperature difference, dimer window, and dimer max ratio. When primers included degenerate positions, the limit of complexity used was set to a maximum of 48. Additionally, the melting temperature (Tm) of each primer was calculated using the empirical nearest neighbor model [49]. In total, four pairs of primers were obtained: one pair for the 1st round of the nested-PCR, which, a priori, would allow the amplification of part of the hexon-coding region of all European HAdV species and types included in the dataset, as well as three pairs for 2nd round primers designed to tentatively amplify the hexon region of HAdV-A and -F, -B and -E, and -D species (Table 1).

### 2.4. Nested Touch-Down PCR Assays

For the partial amplification of the HAdV hexon gene, three nested touch-down PCR protocols were developed: one for the amplification of HAdV-A and -F, one for the amplification of HAdV-B and -E, and another for the amplification of HAdV-D.

The thermal profile used in the 1st round of PCR reactions included 3 min at 95 °C, 10 cycles of 30 s at 95 °C, 30 s at 55 °C (with a decrease of 1 °C per cycle), 1 min and 15 s at 72 °C, 30 cycles of 30 s at 95 °C, 30 s at 45 °C, 1 min and 15 s at 72 °C, and a final extension step of 7 min at 72 °C. Then, the obtained product was used as a template for the 2nd round where the cycling conditions used included 3 min at 95 °C, 10 cycles of 30 s at 95 °C, 30 s at 53 °C (screening for the presence of HAdV-A and -F) or 57 °C (detection of -B, -E, and -D), again with 1 °C decrease per cycle during the first 10 cycles, 1 min and 15 s at 72 °C, 40 cycles of 30 s at 95 °C, 30 s at 43 °C (for screening of -A and -F) or 47 °C (for screening of -B, -E and -D), 1 min and 15 s at 72 °C, and a final extension step of 7 min at 72 °C. All the amplification steps were performed on a T3000 thermocycler (Analytik Jena GmbH, Jena, Germany). In addition, the amplification reactions carried out using the DNA extracted from the environmental samples were performed with the Supreme NZYTaq II Green Master Mix (NZYTech, Lisboa, Portugal), while the NZYTaq II 2x Green Master Mix (NZYTech, Portugal) was used for the reactions with the DNA extracted from the wastewater samples.

After the 2nd round of amplification, the PCR products were analyzed by gel electrophoresis on a 1% agarose gel. The correctly sized amplicons obtained were purified from the agarose gel using the Zymoclean^TM^ Gel DNA Recovery Kit (Zymo Research, Irvine, CA, USA) following the manufacturer’s instructions, and then either directly sequenced (Illumina^®^) or cloned in a plasmid and sequenced by the Sanger method ( STAB VIDA, Lda., Caparica, Portugal).

### 2.5. DNA Cloning and Sanger Sequencing

For DNA cloning, a similar strategy described by Avellón et al. [50] was followed. Briefly, the purified amplicons were cloned using pGEM^®^-T Easy Vector System I Kit (Promega, Madison, WI, USA) and NZYStar chemically competent *Escherichia coli* cells (NZYTech, Portugal). After transformation, individual white clones identified by the absence of α-complementation of the *lacZ*Δ*M15* mutation were selected, and their plasmid DNA was extracted using the NZYMiniprep kit (NZYTech, Portugal).

A minimum of one and a maximum of eight recombinant plasmids were sequenced for each sample using the Sanger method (STAB VIDA, Lda., Portugal).

### 2.6. NGS Sequencing

The NGS sequencing was performed as described previously by Lun et al. [26] with some modifications. So, briefly, the purified DNA products were quantified using the Qubit^®^ dsDNA Broad-Range Assay Kit (Invitrogen, Waltham, MA, USA), and their concentration was adjusted to be within 1–20 ng/µL range, using 0.1X Tris-EDTA (TE) buffer. Afterward, all DNA samples were fragmented by sonication using the Bioruptor^®^ Plus (Diagenode, Liège, Belgium) by applying six repetitions of five cycles of 30 s and 90 s ON and OFF, respectively.

Next-Generation Sequencing libraries were prepared using the NEBNext^®^ Ultra^TM^ II Library Prep Kit for Illumina^®^ (New England Biolabs, Ipswich, MA, USA) and quantified with the NEBNext^®^ Library Quant Kit for Illumina^®^ (New England Biolabs, USA). The DNA fragment sizes were determined using the Agilent High Sensitivity DNA reagent kit (Agilent Technologies, Santa Clara, CA, USA), and diluted in 10 mM Tris-HCl. A 2 nM pooled library was created by combining each diluted library. The libraries pool was normalized according to MiSeq System Denature and Dilute Libraries Guide (Illumina, Lisboa, Portugal) following protocol A—standard normalization method, and using PhiX174 DNA as an internal control of the reaction.

The MiSeq Reagent V2 300 cycle kit (Illumina^®^, San Diego, CA, USA) was used for paired-end sequencing on the MiSeq platform (Illumina^®^, USA).

### 2.7. Nucleotide Sequences Analysis

The sequences obtained by the Sanger method (*n* = 60) were edited with the Trim Ends tool of the Geneious Prime^®^ program (version 2022.1.1) using the error probability limit of 0.001. Then, a taxonomic identification, based on the analysis of the cleaned sequences, was performed with the Nucleotide Basic Local Alignment Search (BLASTn) using the MegaBLAST option. Those sequences identified as not corresponding to AdV (*n* = 19) were not further analyzed and discarded.

The sequences obtained by NGS were edited with the OmicsBox program (version 2.1.14). The FASTQ Preprocessing tool was used for the removal of adapters and contaminating sequences, the trimming of low-quality bases, and the filtering of short and low-quality reads. Nucleotide sequence contigs were obtained using metaSPAdes, then OmicsBox metagenomic gene prediction tool was used, followed by a BLASTn search. From the annotated and mapped BLASTn results, nodes whose sequence description did not contain “hexon” and those corresponding to sequences smaller than 350 bp were excluded.

All the HAdV nucleotide sequences obtained were deposited in the GenBank/ENA/EMBL public genomic databases under the accession numbers OP605769–OP605897.

### 2.8. Phylogenetic Analysis

The taxonomical identification performed by BLASTn was confirmed by phylogenetic analysis for all Sanger sequences (*n* = 41). For sequences obtained using NGS, phylogenetic analysis was performed only for those with an unidentified type of HAdV (*n* = 49) by searching the genomic databases for homologous sequences.

Phylogenetic inference analyses were carried out using nucleotide sequence datasets constructed with >350 bp hexon gene sequences available in the GenBank nucleotide sequence database, and the appropriate evolution model was determined for each one of the datasets with the Model Selection feature of IQ TREE Web Server [51], considering the Akaike Information Criterion. Maximum likelihood phylogenetic trees were constructed using the Tree Inference feature, also implemented in the IQ TREE Web Server, as previously described by Filipa-Silva et al. [52]. The topological stability of the trees was assessed using both the ultrafast bootstrap option with 1000 data resamplings, and 1000 iterations of the approximate likelihood–ratio test (aLRT). Values > 75% (expressed as a percentage of the total number of replicates) were considered as relevant for both tests.

## 3. Results

### 3.1. HAdV Screening in the Collected Water Samples

Of the 72 PCR amplifications performed, 33 were considered successful, i.e., a fragment of the expected size was observed on the agarose gel. The amplification patterns for each sample ranged from a unique fragment to several of them (data not shown). In addition, a comparison of the performance of a conventional nested versus touch-down PCR was made (data not shown). While with conventional PCR no amplification products were obtained, those amplifications considered successful resulted from the use of the nested touch-down PCR approach.

Considering the 24 water samples under study, 14 (58.3%), including eight WWTP and six environmental, revealed the presence of HAdV-A and/or -F. On the other hand, the presence of HAdV-B and/or -E genomic sequences were confirmed in 12 (50%) of the samples under analysis, corresponding to three environmental and nine WWTP water samples. For the HAdV-D PCR protocol, an amplicon with the expected molecular weight was observed in seven (29%) samples, concerning one environmental and six WWTP water samples.

Considering that some of the WWTP sites were sampled more than once, in different years or seasons, and that the viral identification results were reproducible regardless of their collection date, whenever multiple samples were available for a given WWTP, only one of those samples was selected for viral characterization through both molecular cloning in a vector (followed by Sanger sequencing) and NGS sequencing. Therefore, 25 amplicons were used, corresponding to 11, 9, and 5 DNA fragments resulting from PCR A + F, B + E, and D protocols, respectively.

A total of 21 (out of 25) amplicons were purified to proceed with the cloning method. The remaining four, due to their weak amplification yield (despite multiple amplification attempts), were directly analyzed by NGS, along with 16 of the purified products.

### 3.2. Genetic Characterization of AdV, as Defined by Molecular Cloning Followed by Sanger Sequencing

Out of a total of 28 sequences expected to correspond to HAdV-A or -F (considering the obtained amplification results), 11 of them (from WWTP C, Fogueteiro, Sado River–Industrial Area of Setúbal, Lizandro River, Ribeira de Caparide, and Trancão River) showed either high similarity with non-AdV sequences or their origin could not be suggested (as they were not highly similar to any sequence in the database). Unexpectedly, one sequence (from the WWTP B2 sample) was identified as corresponding to the HAdV-D species. From the remaining sequences, 11 showed high similarity with HAdV-A or -F, and five were recognized only as HAdV, as their identity could not be unambiguously established based on sequence-similarity search results. From the recombinant plasmids expected to have a HAdV-B or -E specific insert, 11 were analyzed, and the taxonomic identification of their inserts’ sequences unexpectedly revealed similarity with HAdV-F and -D. For the 20 recombinant plasmids expected to have a HAdV-D specific insert, eight (from the WWTP C sample) showed similarity with non-AdV sequences. Moreover, and again against what was expected, six of them were identified as HAdV-C and only two as HAdV-D.

To confirm the virus identification results obtained using sequence similarity searches, phylogenetic trees were constructed with the sequences suggested to originate from an HAdV or at least AdV (*n* = 41). From this analysis (data not shown) five sequences (from Ribeira do Carenque and Corroios samples) stood out as a monophyletic tree outgroup. Nevertheless, phylogenetic tree reconstruction using hexon gene sequences from non-human AdV, associated them with murine mastadenovirus 2 (MAdV-2).

**Figure 2:**
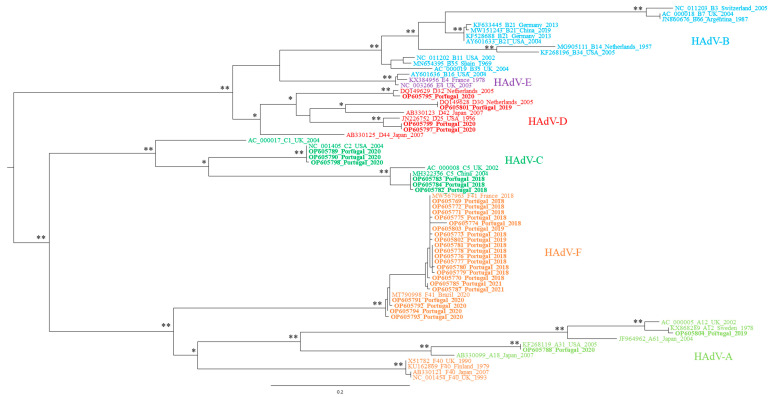
Maximum likelihood phylogenetic tree of HAdV hexon capsid gene sequences, including 33 Portuguese sequences obtained after molecular cloning/Sanger sequencing. Each sequence is identified by its accession number, HAdV type, country of origin, and year. The number of “*” indicates the number of methods that support the demonstrated topology, considering 75% (of the total number of data resamplings) and above as relevant for aLRT and bootstrap values. Each HAdV species is indicated in a different color: A—light green, B—blue, C—dark green, D—red, E—purple, and F—orange.

**Figure 3:**
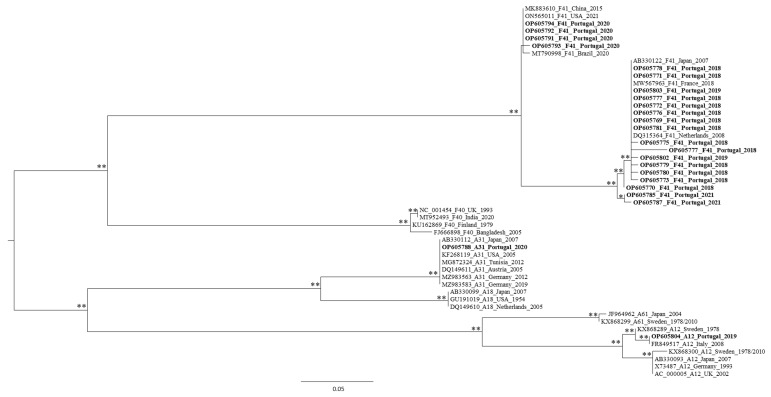
Maximum likelihood phylogenetic tree of HAdV-A and -F hexon capsid gene sequences, including 23 Portuguese sequences obtained by molecular cloning/Sanger sequencing. Each sequence is identified by its accession number, HAdV type, country of origin, and year. The number of “*” indicates the number of methods that support the demonstrated topology, considering 75% (of the total number of data resamplings) and above as relevant for aLRT and bootstrap values.

A phylogenetic tree was tentatively constructed with the remaining 36 HAdV sequences obtained, however three of them (from the Alhos Vedros and WWTP F samples) were also analyzed individually (owing to their relatively smaller size), again by phylogenetic analysis (data not shown). These sequences were shown to cluster along with the HadV-F41 viral references, while the remaining two, from the WWTP F, were associated with the HadV-D species radiation. A phylogenetic tree was then constructed with the remaining 33 sequences (Figure 2), and most of them were found to be a part of the HadV F species. Moreover, since we aimed to evaluate the molecular diversity of HadV types circulating in LMA, specific phylogenetic trees were constructed for each species (HadV-A and -F, -C, and -D) to assign one viral type to each sequence (Figure 3 for HadV-A and -F species, Supplementary Figures S1 and S2 for -C and -D species, respectively). In line with the similarity search results obtained, some of the HadV sequences that had been amplified using PCR A + F primers clustered among HadV-D species, while others amplified with PCR B + E were shown to cluster with HadV-F41, -D32 types. Finally, a few of those amplified by PCR D clustered with HadV-C2, and -C5 types. These phylogenetic reconstruction results corroborate the BLASTn results, suggesting that the primers used were not 100% specific for the HadV species they were designed to target.

**Figure 4:**
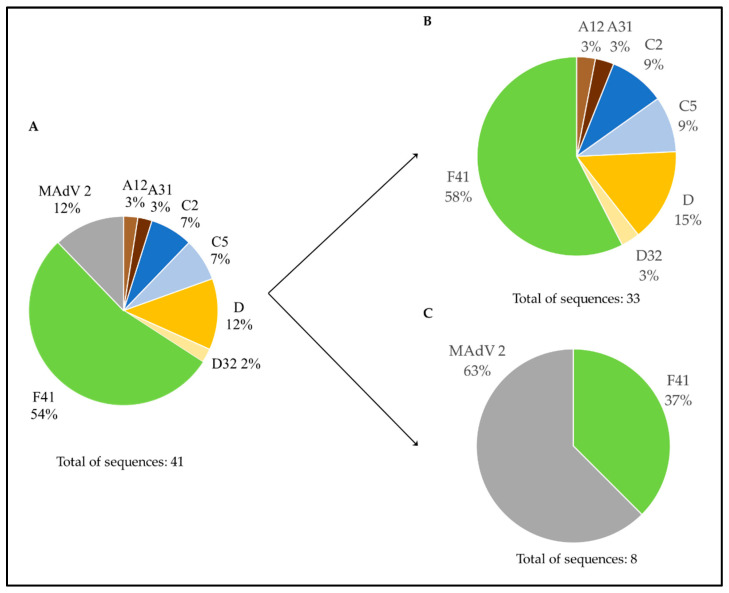
Graphical distribution of HAdV types assigned to the Portuguese sequences obtained from combining molecular cloning and Sanger sequencing. (**A**) HAdV types considering both water matrices. HAdV types associated with WWTPs (**B**) and environmental samples (**C**). Each color represents one viral type/species: A12—light brown, A31—dark brown, C2—dark blue, C5—light blue, D—yellow, D32—light yellow, F41—green, murine mastadenovirus (MAdV-2) 2—gray. “WWTP” stands for “wastewater treatment plant”.

Figure 4 summarizes the distribution of the types assigned to the 41 Portuguese sequences obtained from the molecular cloning method followed by Sanger sequencing. When observing the distribution of each viral type, HAdV-F41 accounted for 54% of the total number of sequences. Additionally, Figure 4 presents the distribution of the obtained HAdV types according to the type of sample (nine WWTP and three environmental). The greatest diversity of HAdV types was observed in WWTP samples.

### 3.3. Genetic Characterization of AdV Sequences as Defined by the Analysis of AdV Amplicons Using an NGS (Illumina) Approach

**Figure 5:**
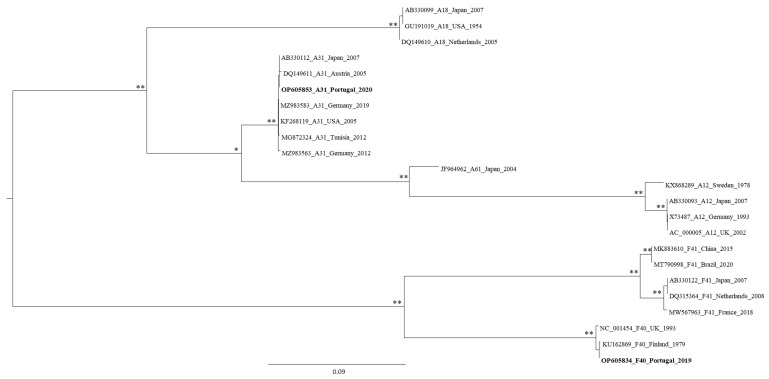
Maximum likelihood phylogenetic tree by of HAdV-A and -F hexon capsid gene sequences, including two Portuguese sequences obtained by NGS. Each sequence is identified by its accession number, HAdV type, country of origin, and year. The number of “*” indicates the number of methods that support the demonstrated topology, considering 75% (of the total number of data resamplings) and above as relevant for aLRT and bootstrap values. Altogether, the analysis of all of the contigs obtained revealed the presence of AdV genomes in all samples analyzed (14 WWTP and six environmental). The great majority (*n* = 93; 73%) of the 127 contigs mentioned were characterized as HAdV, and 34 (27%) were suggested as related to MAdV-2. Figure 6 presents all HAdV types assigned (combining BLASTn and phylogenetic reconstruction results), and also the distribution of the AdVs according to the type of sample analyzed.

High throughput sequencing of a pooled library of AdV amplicons revealed 127 AdV hexon-specific contigs larger than 350 bp. The sequences for which a viral type classification could not be achieved using only BLASTn-driven database sequence searches were divided into three datasets according to the species classification suggested (HAdV-A, -F, -C, or -D). The distribution of 11 of the analyzed sequences within the HAdV species is presented in Figure 5 for -A and -F, and Supplementary Figures S3 and S4 for -C and -D species, respectively.

Additional phylogenetic trees were also constructed to confirm those sequence similarity search results for which BLASTn identity values were below 95%. In this case, 34 were shown to be closely related to MAdV-2, three others clustered with HAdV-D42, while a single one clustered with HAdV-D26 (data not shown).

## 4. Discussion

This study aimed at the genetic characterization of HAdV sequences obtained from water samples collected in Portugal. In particular, for logistic reasons and because Lisbon is the largest city hub in the country, this report is based on the analysis of water samples collected in the so-called Lisbon Metropolitan Area, including Lisbon and its surroundings. Since the HAdV genome is very stable and occurs in higher frequency and abundance in water when compared to other enteric viruses [1,14], the detection of HAdVs in many of the water samples studied was expected, especially in those corresponding to, or clearly contaminated with, residual water. Not surprisingly, the highest AdV hexon-sequence amplification rate was obtained for the WWTP influent samples when compared with the environmental ones used, corroborating previous studies where the presence of HAdV in untreated sewage was frequently disclosed [24,26,32,53,54].

Although the environmental sampling points were selected based on their proximity to possible fecal sources, the difference in the amplification efficiency regarding both types of water samples was expectable, especially given the expected decrease in the HAdV titer in the environmental samples by dilution effect. Recently, Monteiro et al. [55] showed that rainfall incidence affects HAdV levels, and even though AdV are supposed to be present throughout the year [13,55], all environmental samples were collected during the autumn season. Therefore, the hypothesis that the rain could have diluted the total viral input, resulting in a decrease in the HAdV titter, cannot be ignored. Furthermore, unlike most of the environmental samples where HAdV DNA was amplified, some others corresponded to estuary river samples (Tejo and Sado basins) and were collected relatively close to the river mouth and therefore close to the Atlantic Ocean. In this case, the diluting impact of a large body of water under the influence of tides cannot be neglected.

On the other hand, the inability to detect an HAdV genome in a given sample can also result from the workflow applied to its processing. In fact, and as expected, when combining molecular cloning and Sanger sequencing, the smaller total number of sequences obtained prevented the detection of viral sequences when many of those analyzed were non-viral in nature. In over one-third of the samples analyzed (7 in 19; 36.8%), extreme situations occurred for which no HAdV DNA sequences were identified using this strategy, regardless of the nature (WWTP or environmental) of the water matrix used. Thus, in this case, the “classical” workflow (cloning followed by sequencing) was not able to confirm (considering the relatively small number of clones analyzed) the result suggested by the PCR screening step. Given that there is a high probability of finding HAdV DNA at least in WWTP samples, this is quite an odd occurrence. For this reason, in the genetic characterization endeavor based on NGS, some of the samples for which the molecular cloning/Sanger sequencing strategy failed to reveal the presence of HAdV DNA, were included (WWTP C, Ribeira das Vinhas, Ribeira de Caparide, and Trancão River). As expected, the presence of HAdV sequences was revealed in all of them. Therefore, despite the technical simplicity and affordability of the “classical” molecular cloning-sequencing approach, this strategy introduces qualitative biases in the results.

Unsurprisingly, the more technically complex and expensive NGS-based sequencing approach clearly overshadowed the performance of the “classical” strategy with its high throughput sequencing capacity, which becomes especially relevant when the intended genetic analysis is based on complex samples such as those coming from WWTPs. Therefore, given that traditional cloning and Sanger sequencing-based protocols are less sensitive than mass-parallel sequencing, their sole utilization may inaccurately reflect the circulation of HAdVs in a given environment. As also suggested by Iaconelli et al. [24], these results show that a more comprehensive picture of the distribution of HAdV types in water can be predicted from water monitorization by NGS technologies, in particular using the Illumina MiSeq system, which is the approach most used for pathogen surveillance [56].

Regarding the diversity of HAdV types in LMA, the assignment of one viral type to each sequence was achieved for almost all sequences, revealing the genetic variability of the Portuguese sequences, except for some of the HAdV-D species. As noted by Amdiouni et al. [57] and Casas et al. [58], the viral types within the HAdV-D species share a high sequence identity, making it very difficult to distinguish among them unambiguously.

Over time, several studies have reported the detection of HAdV in different water matrices, such as rivers [29,33,54,59,60], lakes [61], and beaches [62], as well as in wastewater samples collected in several geographical locations around the world [15,24,26,39,53,59], being HAdV-F41 one of the most commonly detected HAdV types [12,14,63]. In this study, both approaches allowed the identification of several HAdV types in LMA. Our findings support previous studies, since HAdV-A12, -A31, -B3, -C1, -C2, -D26, -D37, -D42, -F40, and -F41 types were also reported worldwide, including in European countries located closer to Portugal (such as Italy and Spain) [12,14,15,23,24,25,26,33,40,61,63]. Additionally, the results here obtained are in accordance with the literature, highlighting the great prevalence of HAdV-F41. Although all HAdV types (enteric and non-enteric) can be excreted and found in high concentrations in residual water [24,29,30], the identification of other types of HAdV by the molecular cloning method was surprising given that not all HAdV types were targeted by the nested-PCR protocols used. The HAdV-C species, which was not considered for primer design, is one of such cases.

HAdV are enteric viruses and, consequently, are directly excreted into sewage systems. For this simple reason, finding a greater diversity of viral types when assessing raw sewage samples from WWTPs was expected. On the other hand, fecal contamination of environmental waters refers to point sources, such as the discharge of industrial/municipal/domestic wastewater, and non-point sources including storm/urban/agricultural water runoff [64]. The lower diversity of HAdV hexon sequences detected in the environment may suggest that in the case of sewage leakage, the majority of HAdV types were already eliminated by WWTP treatments, the only ones remaining having a higher propensity to persist in the aquatic environment and/or are present in higher titers, or are associated with mammals frequently inhabiting sewage-contaminated water sources (e.g., rats and mice; see below). HAdVs are more resistant to chemical and physical agents than other viruses and fecal indicator bacteria, and this is particularly true regarding HAdV-F [13]. For example, the predominance of HAdV-F41 in the environment might be explained by their greater ability to persist in natural environments when compared to other types, as suggested by Fong et al. [60]. Rafie et al. [65] demonstrated that these viruses possess a capsid whose structure is not modified by stomach pH, and whose virion surface has substantial changes when compared to non-enteric HAdVs (e.g., HAdV-F41 has fewer pH-dependent residues exposed on the surface of its capsid). As a result, HAdV-F41 seems to be adapted to the specific conditions in the gastrointestinal tract, perhaps also resulting in a higher resistance when released outside their hosts.

The presence of AdV genomes in rodents has been previously reported in other studies [66,67,68,69]. In particular, two AdV types have already been isolated from house mice (*Mus musculus*): MAdV-1 and MAdV-2, with the latter being responsible for infections of the gastrointestinal tract [66]. In addition, some serological studies have suggested that AdV infections in rodents may be frequent [67]. Although the identification of the MAdV viral genome in this study was only supported by phylogenetic reconstruction (since BLASTn identity values were below 95%), its possible detection in the samples studied was not surprising, considering that these animals can also contaminate the water with their excreta. Therefore, even when using primers designed to detect specific species of HAdVs and touch-down amplification protocols, working with highly complex samples not only failed in the exclusive amplification of HAdV homologous targets, but also allowed the detection of non-targeted HAdV, as well as MAdV.

The data from both approaches applied in this study provide important information about the distribution of HAdV in the Portuguese population. The results reported here disclose the circulation of HAdV-A12, -A31, -B3, -C1, -C2, -C5, -D8, -D15, -D22, -D26, -D32, -D37, -D42, -D45, -D56, -F40, and -F41 types in LMA, between 2018 and 2021, either in waste or environmental waters. However, finding a high diversity of HAdV types is not, a priori, a concern since their presence is not necessarily synonymous with disease. In other words, these viruses can persist in the infected population, not inducing any kind of disease, and remaining silent. Of all types detected in this study, HAdV-B3, -C1, -C2, -C5, and -F41 were frequently reported in the literature as associated with human disease [23,25]. However, their prevalence was low (1–2% in both approaches), except for -F41. Although HAdVs are often efficiently eliminated as a result of the WWTP secondary treatment, with a considerable reduction in the titer of infectious viruses [26], treated wastewater can contaminate surface water. Consequently, the relatively high detection of HAdV-F41, which is a common cause of severe gastroenteritis and diarrhea-related death in young children across the world [65,70], can be a concern. In a similar study, Lun et al. [26] demonstrated that HAdV-F41 was the most detected HAdV type in clinical samples. Although only water samples were analyzed in this study, it is not surprising that HAdV-F41 might have been associated with disease in some of the population from LMA. HAdV detection in water samples highlights the effectiveness of using them for virus surveillance [26], but more studies must be performed to evaluate the infectivity of these viruses, and subsequently, to assess their risk to public health.

According to several studies that have proposed the use of HAdV as an indication of viral contamination in water [9,13,14,15,16], this study demonstrated (through the detection of HAdV genomes in wastewater and environmental samples), that these viruses can be useful to monitor fecal contamination. The presence of HAdV DNA in the environmental samples suggests that the Trancão River, the creeks, and the ditches analyzed from LMA were, at some point, contaminated with fecal matter, probably due to the inefficiency of the conventional treatments applied by WWTPs, or due to direct contamination from sewage. This contamination can represent a public health problem. Moreover, the Corroios and Alhos Vedros ditches were chosen due to the complaint of the nearest residents, regarding the smell after sewage discharges. Before the water collection, the Corroios ditch was cleaned; however, HAdV DNA was still detected (and possibly MAdV DNA). This underlines the extreme resistance of these viruses to environmental degradation and cleaning processes, especially the resistance of its genome, as revealed in other studies [1,14,71], which also indicates that the contamination source was not eradicated.

## 5. Conclusions

To our knowledge, only a restricted number of studies have surveyed the distribution and assessed the titer, but not the diversity, of HAdV types in Portuguese waters. In contrast, this study focused on the molecular diversity of HAdV types circulating in LMA through the analysis of raw sewage (i.e., influent) and environmental water samples.

The results showed the presence of viral genomes related to HAdV-A, -B, -C, -D, and -F species, with HAdV-F41 being the most predominant viral type present, corroborating literature reports worldwide. In addition to the identification of a large diversity of HAdV species and types, sequences possibly related to murine AdV-2 have also been described.

Along with other studies, this work uncovered the occurrence of some HAdV types in wastewater, also highlighting the presence of fecal contamination in environmental waters. This is probably due to the inefficiency of the conventional applied treatments by WWTPs or to direct contamination with raw or partially treated sewage discharges from domestic or small industries.

Furthermore, the assessment of the diversity of this virus (through the study of its genomes) provides important information about the patterns of its molecular epidemiological distribution in the population.

## Figures and Tables

**Figure 6:**
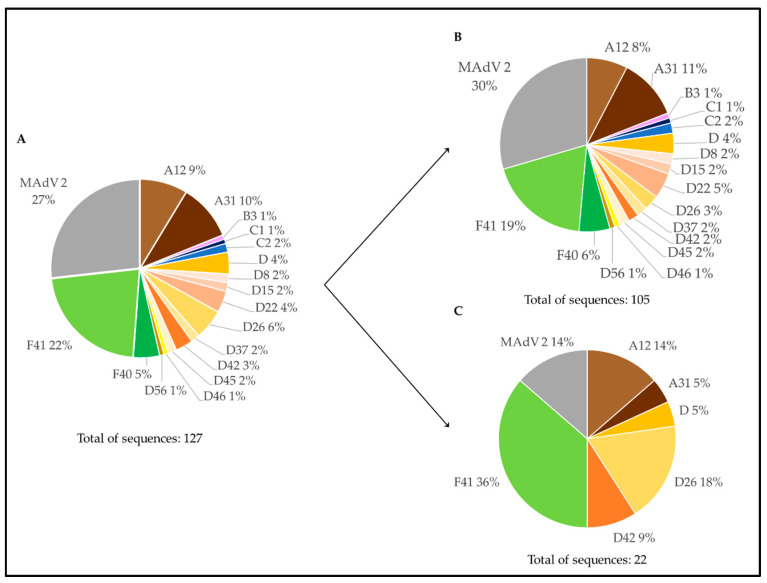
Graphical distribution of HAdV types assigned to the Portuguese sequences obtained by NGS, including the ones also analyzed by phylogeny. (**A**) HAdV types considering both water matrices. HAdV types in WWTP (**B**) and environmental (**C**) samples. Each color represents one viral type/species: A12—light brown, A31—dark brown, B3—light purple, C1—dark blue, C2—light blue, D—a variation of yellow and orange, F40—dark green, F41—light green, murine mastadenovirus (MAdV) 2—gray. “WWTP” stands for “wastewater treatment plant”.

**Table 1:** List of primers designed and used for the HAdV screening in the collected water samples, corresponding complexity limit, melting temperature (Tm), and size of the expected amplicon.

PCR Round	Primer Name	Primer Sequence (5′–3′)	Complexity	Tm (Mean) (°C)	Amplicon (bp)
1st	FW universal	CTRGCYGTGGGYGAYAACMG	32	63.94	1615
	RV universal	GAYTGRTCRTTGGTRTTCRTT	32	56.50	
2nd	FW A + F	TAYCARCCVGARCCKCAAGT	48	62.11	1108
	RV A + F	AAGTTCCAYTCRTAVGTGTA	12	54.75	
	FW B + E	GTRGGCGACAACMGHGTGCT	12	65.69	702
	RV B + E	AAGCCAATGTARTTGGGTCTGTT	2	61.51	
	FW D	TTCAAACCCTACTCGGGCAC	1	61.83	1339
	RV D	TGATGGCAAAGAACTTTTGGGGC	1	63.76	

H (A, C, or T), K (G or T), M (A or C), R (A or G), S (C or G), Y (C or T), V (A, C or G). “°C” indicates degrees Celsius, “bp” stands for “base pairs”, “FW” for “forward”, and “RV” for “reverse”. This work is the reference for all the primers listed.

## Data Availability

Data are contained within the article or Supplementary Materials.

## Supplementary Materials

The following supporting information can be downloaded at: https://www.mdpi.com/article/10.3390/microorganisms10122443/s1, Figure S1: Maximum likelihood phylogenetic tree of HAdV-C hexon capsid gene sequences, including six Portuguese sequences obtained by molecular cloning/Sanger sequencing. Each sequence is identified by its accession number, HAdV type, country of origin, and year. The number of “*” indicates the number of methods that support the demonstrated topology, considering 75% (of the total number of data resamplings) and above as relevant for aLRT and bootstrap values. Figure S2. Maximum likelihood phylogenetic tree of HAdV-D hexon capsid gene sequences, including three Portuguese sequences obtained by molecular cloning/Sanger sequencing. Each sequence is identified by its accession number, HAdV type, country of origin, and year. The number of “*” indicates the number of methods that support the demonstrated topology, considering 75% (of the total number of data resamplings) and above as relevant for aLRT and bootstrap values. Figure S3. Maximum likelihood phylogenetic tree of HAdV-C hexon capsid gene sequences, including two Portuguese sequences obtained by NGS. Each sequence is identified by its accession number, HAdV type, country of origin, and year. The number of “*” indicates the number of methods that support the demonstrated topology, considering 75% (of the total number of data resamplings) and above as relevant for aLRT and bootstrap values. Figure S4. Maximum likelihood phylogenetic tree of HAdV-D hexon capsid gene sequences, including seven Portuguese sequences obtained by NGS. Each sequence is identified by its accession number, HAdV type, country of origin, and year. The number of “*” indicates the number of methods that support the demonstrated topology, considering 75% (of the total number of data resamplings) and above as relevant for aLRT and bootstrap values.

## References

[1] Verani M., Federigi I., Donzelli G., Cioni L., Carducci A. (2019). Human Adenoviruses as Waterborne Index Pathogens and Their Use for Quantitative Microbial Risk Assessment. Sci. Total Environ..

[2] Calgua B., Fumian T., Rusiñol M., Rodriguez-Manzano J., Mbayed V.A., Bofill-Mas S., Miagostovich M., Girones R. (2013). Detection and Quantification of Classic and Emerging Viruses by Skimmed-Milk Flocculation and PCR in River Water from Two Geographical Areas. Water Res..

[3] Fong T.-T., Lipp E.K. (2005). Enteric Viruses of Humans and Animals in Aquatic Environments: Health Risks, Detection, and Potential Water Quality Assessment Tools. Microbiol. Mol. Biol. Rev..

[4] Hammond P., Suttie M., Lewis V.T., Smith A.P., Singer A.C. (2021). Detection of Untreated Sewage Discharges to Watercourses Using Machine Learning. Npj Clean Water.

[5] Sato T., Qadir M., Yamamoto S., Endo T., Zahoor A. (2013). Global, Regional, and Country Level Need for Data on Wastewater Generation, Treatment, and Use. Agric. Water Manag..

[6] Ullah Bhat S., Qayoom U., Zhang T. (2022). Implications of Sewage Discharge on Freshwater Ecosystems. Sewage-Recent Advances, New Perspectives and Applications.

[7] Almeida P., Albuquerque T., Antunes M., Ferreira A., Pelletier G. (2021). Effects of Wastewater Treatment Plant’s Discharges on a Freshwater Ecosystem—A Case Study on the Ramalhoso River (Portugal). Water Air Soil Pollut..

[8] Opere W.M., John M., Ombori O. (2020). Occurrence of Enteric Viruses in Surface Water and the Relationship with Changes in Season and Physical Water Quality Dynamics. Adv. Virol..

[9] La Rosa G., Pourshaban M., Iaconelli M., Muscillo M. (2010). Quantitative Real-Time PCR of Enteric Viruses in Influent and Effluent Samples from Wastewater Treatment Plants in Italy. Ann. Ist Super. SanItà.

[10] Kokkinos P.A., Ziros P.G., Mpalasopoulou A., Galanis A., Vantarakis A. (2011). Molecular Detection of Multiple Viral Targets in Untreated Urban Sewage from Greece. Virol. J..

[11] Gonzales-Gustavson E., Rusiñol M., Medema G., Calvo M., Girones R. (2019). Quantitative Risk Assessment of Norovirus and Adenovirus for the Use of Reclaimed Water to Irrigate Lettuce in Catalonia. Water Res..

[12] Iaconelli M., Muscillo M., Della Libera S., Fratini M., Meucci L., De Ceglia M., Giacosa D., La Rosa G. (2017). One-Year Surveillance of Human Enteric Viruses in Raw and Treated Wastewaters, Downstream River Waters, and Drinking Waters. Food Environ. Virol..

[13] Hewitt J., Greening G.E., Leonard M., Lewis G.D. (2013). Evaluation of Human Adenovirus and Human Polyomavirus as Indicators of Human Sewage Contamination in the Aquatic Environment. Water Res..

[14] Wyn-Jones A.P., Carducci A., Cook N., D’Agostino M., Divizia M., Fleischer J., Gantzer C., Gawler A., Girones R., Höller C. (2011). Surveillance of Adenoviruses and Noroviruses in European Recreational Waters. Water Res..

[15] Bofill-Mas S., Albinana-Gimenez N., Clemente-Casares P., Hundesa A., Rodriguez-Manzano J., Allard A., Calvo M., Girones R. (2006). Quantification and Stability of Human Adenoviruses and Polyomavirus JCPyV in Wastewater Matrices. Appl. Environ. Microbiol..

[16] Rames E., Roiko A., Stratton H., Macdonald J. (2016). Technical Aspects of Using Human Adenovirus as a Viral Water Quality Indicator. Water Res..

[17] Benkő M., Aoki K., Arnberg N., Davison A.J., Echavarría M., Hess M., Jones M.S., Kaján G.L., Kajon A.E., Mittal S.K. (2022). ICTV Virus Taxonomy Profile: Adenoviridae 2022: This Article Is Part of the ICTV Virus Taxonomy Profiles Collection. J. Gen. Virol..

[18] Maclachlan N.J., Dubovi E.J., Barthold S.W., Swayne D.E., Winton J.R. (2017). Fenner’s Veterinary Virology.

[19] Mennechet F.J.D., Paris O., Ouoba A.R., Salazar Arenas S., Sirima S.B., Takoudjou Dzomo G.R., Diarra A., Traore I.T., Kania D., Eichholz K. (2019). A Review of 65 Years of Human Adenovirus Seroprevalence. Expert Rev. Vaccines.

[20] Gallardo J., Pérez-Illana M., Martín-González N., San Martín C. (2021). Adenovirus Structure: What Is New?. Int. J. Mol. Sci..

[21] Besson S., Vragniau C., Vassal-Stermann E., Dagher M.C., Fender P. (2020). The Adenovirus Dodecahedron: Beyond the Platonic Story. Viruses.

[22] Crenshaw B.J., Jones L.B., Bell C.R., Kumar S., Matthews Q.L. (2019). Perspective on Adenoviruses: Epidemiology, Pathogenicity, and Gene Therapy. Biomedicines.

[23] Lynch J., Kajon A. (2016). Adenovirus: Epidemiology, Global Spread of Novel Serotypes, and Advances in Treatment and Prevention. Semin. Respir. Crit. Care Med..

[24] Iaconelli M., Valdazo-González B., Equestre M., Ciccaglione A.R., Marcantonio C., Della Libera S., La Rosa G. (2017). Molecular Characterization of Human Adenoviruses in Urban Wastewaters Using next Generation and Sanger Sequencing. Water Res..

[25] Allard A., Vantarakis A., Rose J.B., Jiménez Cisneros B., Michigan State University, UNESCO-International Hydrological Programme (2017). Adenoviruses. Water and Sanitation for the 21st Century: Health and Microbiological Aspects of Excreta and Wastewater Management (Global Water Pathogen Project).

[26] Lun J.H., Crosbie N.D., White P.A. (2019). Genetic Diversity and Quantification of Human Mastadenoviruses in Wastewater from Sydney and Melbourne, Australia. Sci. Total Environ..

[27] Radke J.R., Cook J.L. (2018). Human Adenovirus Infections: Update and Consideration of Mechanisms of Viral Persistence. Curr. Opin. Infect. Dis..

[28] Heim A. (2021). Diagnosis, Treatment and Prevention of Virus Infections. Encyclopedia of Virology.

[29] Sedji M.I., Varbanov M., Meo M., Colin M., Mathieu L., Bertrand I. (2018). Quantification of Human Adenovirus and Norovirus in River Water in the North-East of France. Environ. Sci. Pollut. Res..

[30] Mena K.D., Gerba C.P. (2009). Waterborne Adenovirus. Reviews of Environmental Contamination and Toxicology.

[31] Lee S.-H., Kim S.-J. (2002). Detection of Infectious Enteroviruses and Adenoviruses in Tap Water in Urban Areas in Korea. Water Res..

[32] Bisseux M., Colombet J., Mirand A., Roque-Afonso A.-M., Abravanel F., Izopet J., Archimbaud C., Peigue-Lafeuille H., Debroas D., Bailly J.-L. (2018). Monitoring Human Enteric Viruses in Wastewater and Relevance to Infections Encountered in the Clinical Setting: A One-Year Experiment in Central France, 2014 to 2015. Eurosurveillance.

[33] La Rosa G., Sanseverino I., Della Libera S., Iaconelli M., Ferrero V.E.V., Barra Caracciolo A., Lettieri T. (2017). The Impact of Anthropogenic Pressure on the Virological Quality of Water from the Tiber River, Italy. Lett. Appl. Microbiol..

[34] Rusiñol M., Fernandez-Cassi X., Timoneda N., Carratalà A., Abril J.F., Silvera C., Figueras M.J., Gelati E., Rodó X., Kay D. (2015). Evidence of Viral Dissemination and Seasonality in a Mediterranean River Catchment: Implications for Water Pollution Management. J. Environ. Manag..

[35] Grøndahl-Rosado R.C., Yarovitsyna E., Trettenes E., Myrmel M., Robertson L.J. (2014). A One Year Study on the Concentrations of Norovirus and Enteric Adenoviruses in Wastewater and A Surface Drinking Water Source in Norway. Food Environ. Virol..

[36] Maunula L., Söderberg K., Vahtera H., Vuorilehto V.-P., von Bonsdorff C.-H., Valtari M., Laakso T., Lahti K. (2012). Presence of Human Noro- and Adenoviruses in River and Treated Wastewater, a Longitudinal Study and Method Comparison. J. Water Health.

[37] Osuolale O., Okoh A. (2015). Incidence of Human Adenoviruses and Hepatitis A Virus in the Final Effluent of Selected Wastewater Treatment Plants in Eastern Cape Province, South Africa. Virol. J..

[38] Staggemeier R., Heck T.M.S., Demoliner M., Ritzel R.G.F., Röhnelt N.M.S., Girardi V., Venker C.A., Spilki F.R. (2017). Enteric Viruses and Adenovirus Diversity in Waters from 2016 Olympic Venues. Sci. Total Environ..

[39] Quintão T.S.C., Silva F.G., Pereira A.L., Araújo W.N., Oliveira P.M., Souza M.B.L.D., Lamounier T.A., Haddad R. (2021). Detection and Molecular Characterization of Enteric Adenovirus in Treated Wastewater in the Brazilian Federal District. SN Appl. Sci..

[40] Artieda J., Piñeiro L., González M.C., Muñoz M.J., Basterrechea M., Iturzaeta A., Cilla G. (2009). A Swimming Pool-Related Outbreak of Pharyngoconjunctival Fever in Children Due to Adenovirus Type 4, Gipuzkoa, Spain, 2008. Eurosurveillance.

[41] Farkas K., Cooper D.M., McDonald J.E., Malham S.K., de Rougemont A., Jones D.L. (2018). Seasonal and Spatial Dynamics of Enteric Viruses in Wastewater and in Riverine and Estuarine Receiving Waters. Sci. Total Environ..

[42] Rebelo-de-Andrade H., Pereira C., Gíria M., Prudêncio E., Brito M.J., Calé E., Taveira N. (2010). Outbreak of Acute Respiratory Infection among Infants in Lisbon, Portugal, Caused by Human Adenovirus Serotype 3 and a New 7/3 Recombinant Strain. J. Clin. Microbiol..

[43] Gonçalves G., Gouveia E., Mesquita J.R., Almeida A., Ribeiro A., Rocha-Pereira J., São José Nascimento M. (2011). Outbreak of Acute Gastroenteritis Caused by Adenovirus Type 41 in a Kindergarten. Epidemiol. Infect..

[44] Ribeiro J., Ferreira D., Arrabalde C., Almeida S., Baldaque I., Sousa H. (2015). Prevalence of Adenovirus and Rotavirus Infection in Immunocompromised Patients with Acute Gastroenteritis in Portugal. World J. Virol..

[45] Ribeiro A., Ramalheira E., Cunha Â., Gomes N.C.M., Almeida A. (2013). Incidence of Rotavirus and Adenovirus: Detection by Molecular and Immunological Methods in Human Faeces. J. Pure Appl. Microbiol..

[46] Hjelmsø M.H., Hellmér M., Fernandez-Cassi X., Timoneda N., Lukjancenko O., Seidel M., Elsässer D., Aarestrup F.M., Löfström C., Bofill-Mas S. (2017). Evaluation of Methods for the Concentration and Extraction of Viruses from Sewage in the Context of Metagenomic Sequencing. PLoS ONE.

[47] Katoh K., Standley D.M. (2013). MAFFT Multiple Sequence Alignment Software Version 7: Improvements in Performance and Usability. Mol. Biol. Evol..

[48] Castresana J. (2000). Selection of Conserved Blocks from Multiple Alignments for Their Use in Phylogenetic Analysis. Mol. Biol. Evol..

[49] Yoon H., Leitner T. (2015). PrimerDesign-M: A Multiple-Alignment Based Multiple-Primer Design Tool for Walking across Variable Genomes. Bioinformatics.

[50] Avellón A., Pérez P., Aguilar J.C., ortiz de Lejarazu R., Echevarría J.E. (2001). Rapid and Sensitive Diagnosis of Human Adenovirus Infections by a Generic Polymerase Chain Reaction. J. Virol. Methods.

[51] Trifinopoulos J., Nguyen L.-T., von Haeseler A., Minh B.Q. (2016). W-IQ-TREE: A Fast Online Phylogenetic Tool for Maximum Likelihood Analysis. Nucleic Acids Res..

[52] Filipa-Silva A., Parreira R., Martínez-Puchol S., Bofill-Mas S., Barreto Crespo M.T., Nunes M. (2020). The Unexplored Virome of Two Atlantic Coast Fish: Contribution of Next-Generation Sequencing to Fish Virology. Foods.

[53] Ibrahim C., Hassen A., Pothier P., Mejri S., Hammami S. (2018). Molecular Detection and Genotypic Characterization of Enteric Adenoviruses in a Hospital Wastewater. Environ. Sci. Pollut. Res..

[54] Nour I., Hanif A., Zakri A.M., Al-Ashkar I., Alhetheel A., Eifan S. (2021). Human Adenovirus Molecular Characterization in Various Water Environments and Seasonal Impacts in Riyadh, Saudi Arabia. Int. J. Environ. Res. Public Health.

[55] Monteiro S., Ebdon J., Santos R., Taylor H. (2021). Elucidation of Fecal Inputs into the River Tagus Catchment (Portugal) Using Source-Specific Mitochondrial DNA, HAdV, and Phage Markers. Sci. Total Environ..

[56] Berry I.M., Melendrez M.C., Bishop-Lilly K.A., Rutvisuttinunt W., Pollett S., Talundzic E., Morton L., Jarman R.G. (2019). Next Generation Sequencing and Bioinformatics Methodologies for Infectious Disease Research and Public Health: Approaches, Applications, and Considerations for Development of Laboratory Capacity. J. Infect. Dis..

[57] Amdiouni H., Faouzi A., Fariat N., Hassar M., Soukri A., Nourlil J. (2012). Detection and Molecular Identification of Human Adenoviruses and Enteroviruses in Wastewater from Morocco: Molecular Identification of HAdV and EV. Lett. Appl. Microbiol..

[58] Casas I., Avellon A., Mosquera M., Jabado O., Echevarria J.E., Campos R.H., Rewers M., Perez-Breña P., Lipkin W.I., Palacios G. (2005). Molecular Identification of Adenoviruses in Clinical Samples by Analyzing a Partial Hexon Genomic Region. J. Clin. Microbiol..

[59] Ogorzaly L., Walczak C., Galloux M., Etienne S., Gassilloud B., Cauchie H.-M. (2015). Human Adenovirus Diversity in Water Samples Using a Next-Generation Amplicon Sequencing Approach. Food Environ. Virol..

[60] Fong T.-T., Phanikumar M.S., Xagoraraki I., Rose J.B. (2010). Quantitative Detection of Human Adenoviruses in Wastewater and Combined Sewer Overflows Influencing a Michigan River. Appl. Environ. Microbiol..

[61] Nagarajan V., Chen J., Hsu B., Hsu G., Wang J., Hussain B. (2021). Prevalence, Distribution, and Genotypes of Adenovirus and Norovirus in the Puzi River and Its Tributaries and the Surrounding Areas in Taiwan. GeoHealth.

[62] Xagoraraki I., Kuo D.H.-W., Wong K., Wong M., Rose J.B. (2007). Occurrence of Human Adenoviruses at Two Recreational Beaches of the Great Lakes. Appl. Environ. Microbiol..

[63] Bofill-Mas S., Calgua B., Clemente-Casares P., La Rosa G., Iaconelli M., Muscillo M., Rutjes S., de Roda Husman A.M., Grunert A., Gräber I. (2010). Quantification of Human Adenoviruses in European Recreational Waters. Food Environ. Virol..

[64] Ahmed W., Goonetilleke A., Gardner T. (2010). Human and Bovine Adenoviruses for the Detection of Source-Specific Fecal Pollution in Coastal Waters in Australia. Water Res..

[65] Rafie K., Lenman A., Fuchs J., Rajan A., Arnberg N., Carlson L.-A. (2021). The Structure of Enteric Human Adenovirus 41—A Leading Cause of Diarrhea in Children. Sci. Adv..

[66] Hemmi S., Vidovszky M.Z., Ruminska J., Ramelli S., Decurtins W., Greber U.F., Harrach B. (2011). Genomic and Phylogenetic Analyses of Murine Adenovirus 2. Virus Res..

[67] Kumakamba C., N’Kawa F., Kingebeni P.M., Losoma J.A., Lukusa I.N., Muyembe F., Mulembakani P., Makuwa M., LeBreton M., Gillis A. (2020). Analysis of Adenovirus DNA Detected in Rodent Species from the Democratic Republic of the Congo Indicates Potentially Novel Adenovirus Types. New Microbes New Infect..

[68] Diffo J., Ndze V.N., Ntumvi N.F., Takuo J.-M., Mouiche M.M.M., Tamoufe U., Nwobegahay J., LeBreton M., Gillis A., Schneider B.S. (2019). DNA of Diverse Adenoviruses Detected in Cameroonian Rodent and Shrew Species. Arch. Virol..

[69] Zheng X., Qiu M., Ke X., Guan W., Li J., Huo S., Chen S., Zhong X., Zhou W., Xiong Y. (2016). Detection of Novel Adenoviruses in Fecal Specimens from Rodents and Shrews in Southern China. Virus Genes.

[70] Rajan A., Palm E., Trulsson F., Mundigl S., Becker M., Persson B.D., Frängsmyr L., Lenman A. (2021). Heparan Sulfate Is a Cellular Receptor for Enteric Human Adenoviruses. Viruses.

[71] Thompson S.S., Jackson J.L., Suva-Castillo M., Yanko W.A., El Jack Z., Kuo J., Chen C.-L., Williams F.P., Schnurr D.P. (2003). Detection of Infectious Human Adenoviruses in Tertiary-Treated and Ultraviolet-Disinfected Wastewater. Water Environ. Res..

