# Supplementary material for: Mastadenovirus Molecular Diversity in Waste and Environmental Waters from the Lisbon Metropolitan Area
Source: Microorganisms. 2022 Dec 10;10(12):2443. doi: 10.3390/microorganisms10122443 (PMC9783802; doi:10.3390/microorganisms10122443)
Supplement: Supplementary file 1 [file microorganisms-10-02443-s001.zip › microorganisms-2064450-supplementary.pdf]

Supplementary Materials

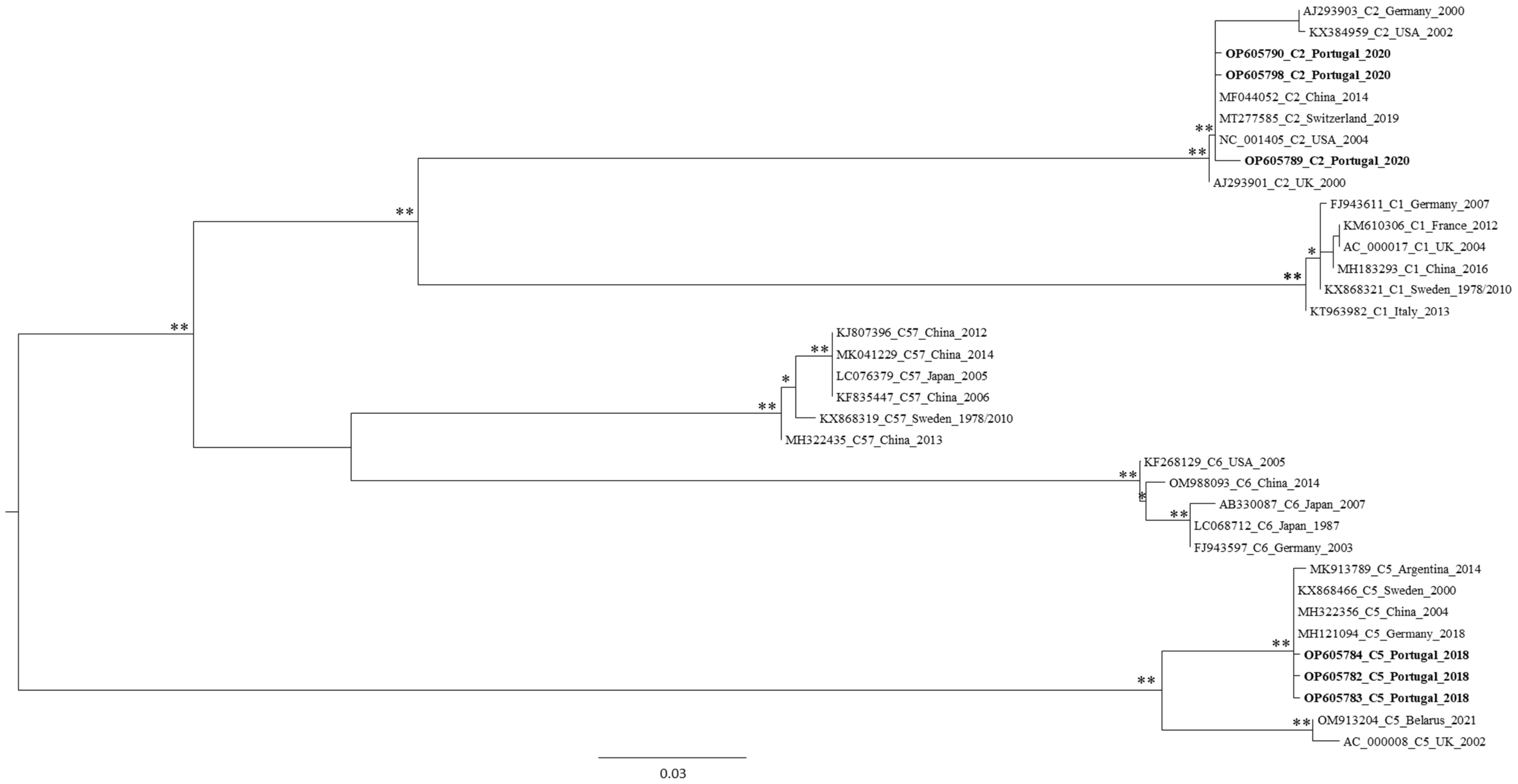

Figure S1. Maximum likelihood phylogenetic tree of HAdV-C hexon capsid gene sequences, including six Portuguese sequences obtained by molecular cloning/Sanger sequencing. Each sequence is identified by its accession number, HAdV type, country of origin, and year. The number of "*" indicates the number of methods that support the demonstrated topology, considering 75% (of the total number of data resamplings) and above as relevant for aLRT and bootstrap values.

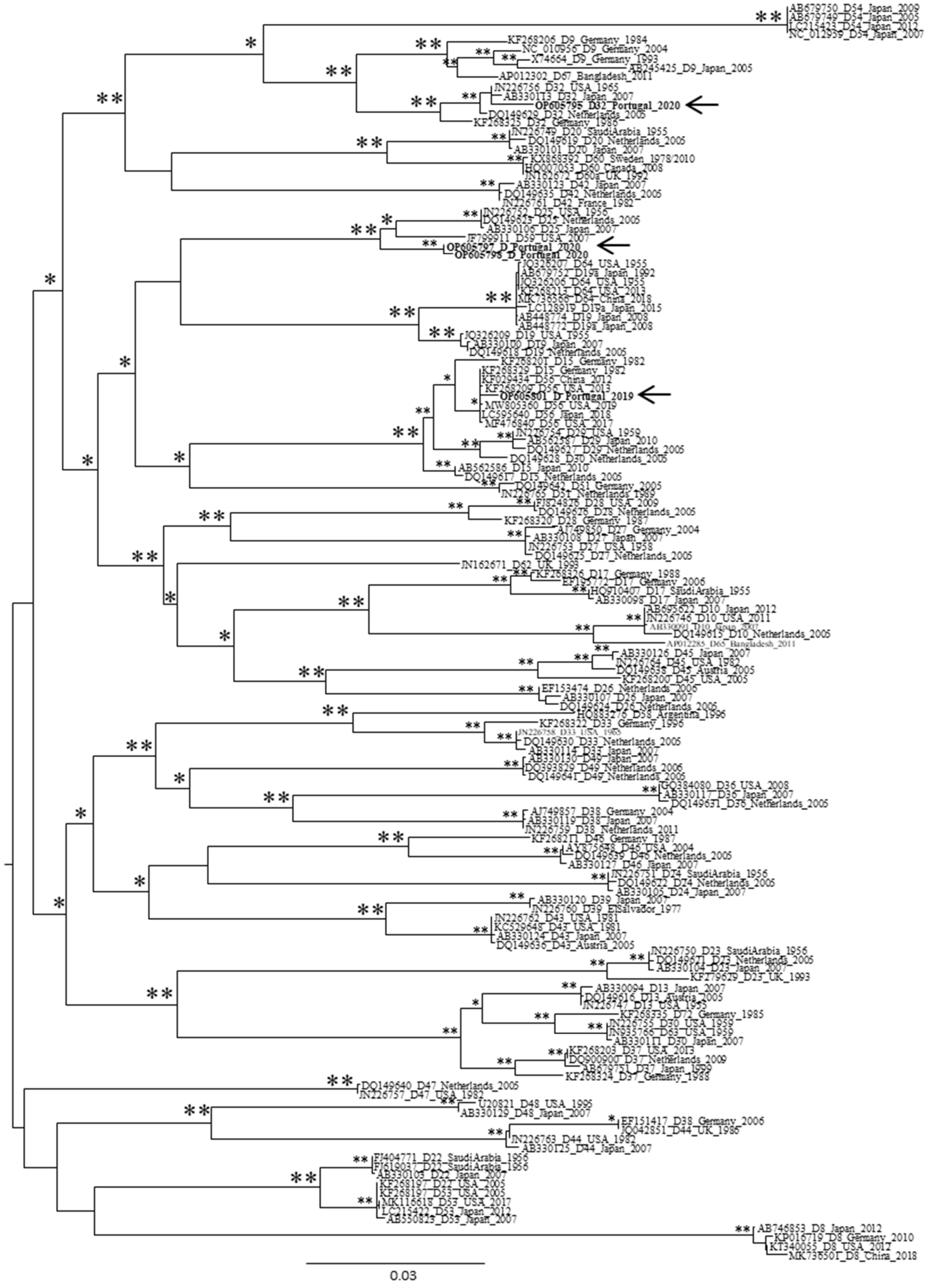

Figure S2. Maximum likelihood phylogenetic tree of HAdV-D hexon capsid gene sequences, including three Portuguese sequences obtained by molecular cloning/Sanger sequencing. Each sequence is identified by its accession number, HAdV type, country of origin, and year. The number of "*" indicates the number of methods that support the demonstrated topology, considering 75% (of the total number of data resamplings) and above as relevant for aLRT and bootstrap values.

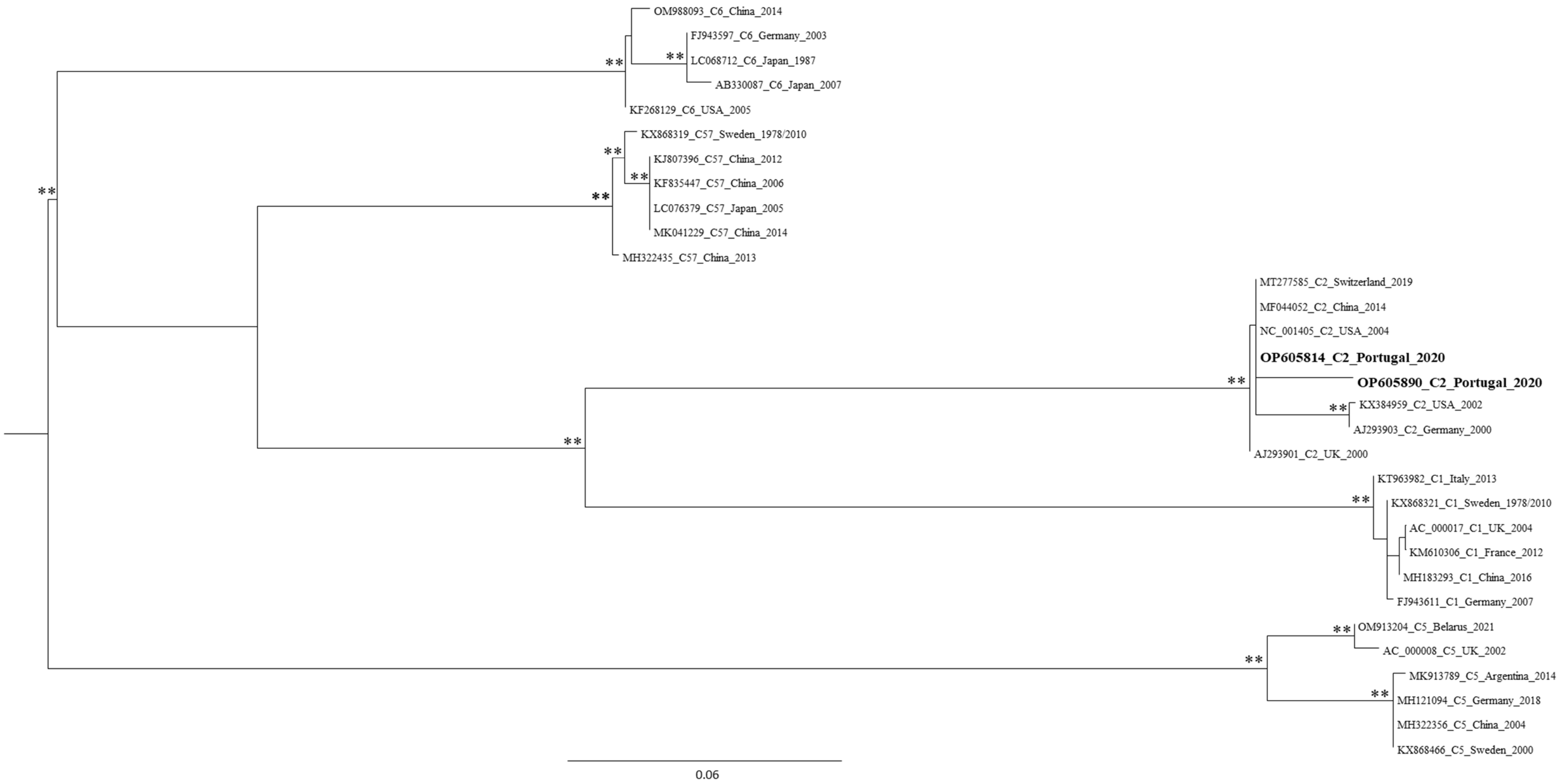

Figure S3. Maximum likelihood phylogenetic tree of HAdV-C hexon capsid gene sequences, including two Portuguese sequences obtained by NGS. Each sequence is identified by its accession number, HAdV type, country of origin, and year. The number of "*" indicates the number of methods that support the demonstrated topology, considering 75% (of the total number of data resamplings) and above as relevant for aLRT and bootstrap values.

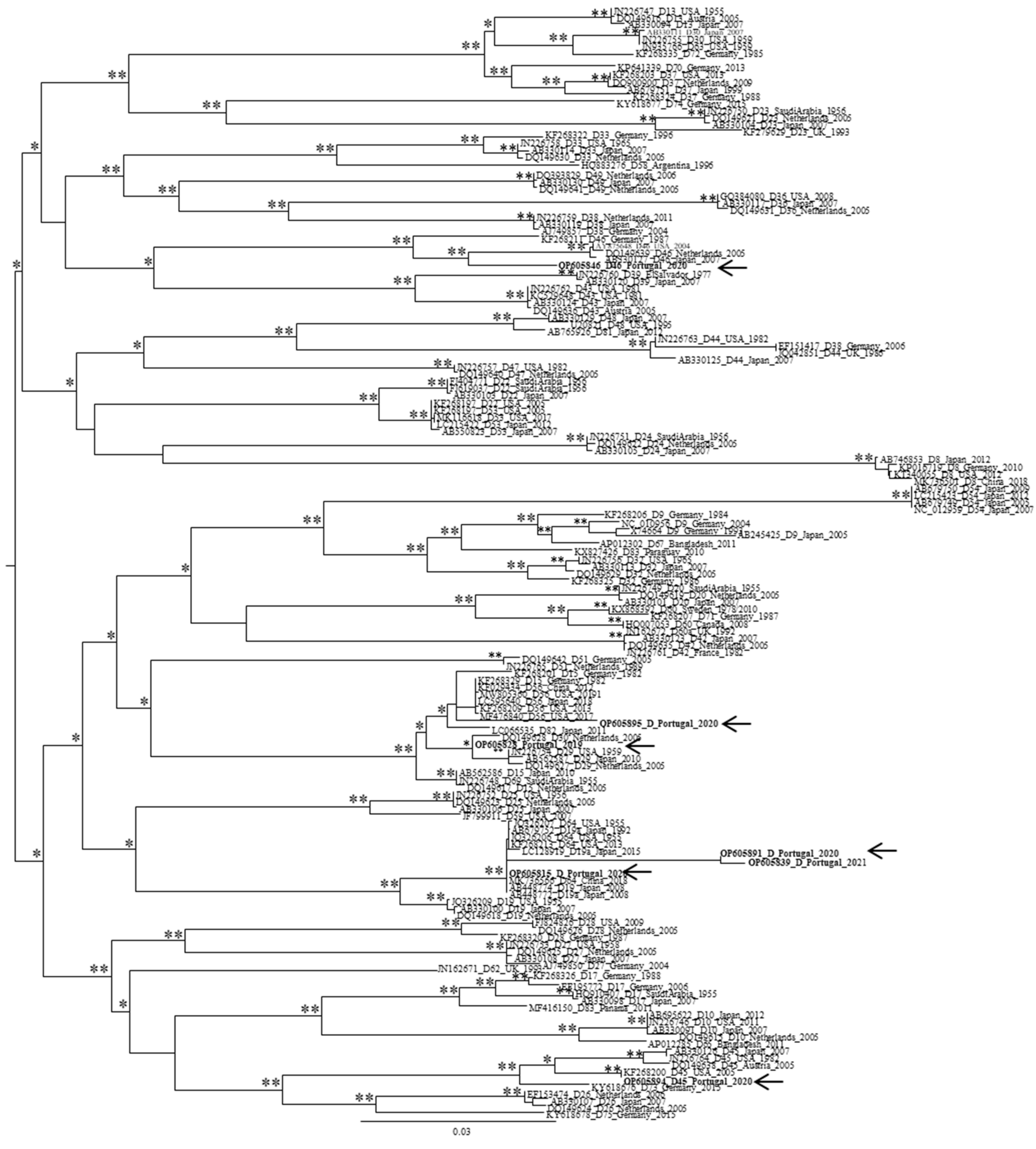

Figure S4. Maximum likelihood phylogenetic tree of HAdV-D hexon capsid gene sequences, including seven Portuguese sequences obtained by NGS. Each sequence is identified by its accession number, HAdV type, country of origin, and year. The number of "*" indicates the number of methods that support the demonstrated topology, considering 75% (of the total number of data resamplings) and above as relevant for aLRT and bootstrap values.
